# Normalization of aberrant resting state functional connectivity in fibromyalgia patients following a three month physical exercise therapy

**DOI:** 10.1016/j.nicl.2015.08.004

**Published:** 2015-08-18

**Authors:** P. Flodin, S. Martinsen, K. Mannerkorpi, M. Löfgren, I. Bileviciute-Ljungar, E. Kosek, P. Fransson

**Affiliations:** a Department of Clinical Neuroscience, Karolinska Institutet, Stockholm, Sweden; b Department of Rheumatology and Inflammation Research, Institute of Medicine, Sahlgrenska Academy, Gothenburg University, Gothenburg, Sweden; c Department of Clinical Sciences, Danderyd Hospital, Karolinska Institutet, Stockholm, Sweden

**Keywords:** FM, fibromyalgia, PAG, periaqueductal grey, FIQ, Fibromyalgia Impact Questionnaire, SF36BP, bodily pain subscale of the Short Form Health Survey, Fibromyalgia, Physical exercise, Resting state fMRI, Functional connectivity

## Abstract

Physical exercise is one of the most efficient interventions to mitigate chronic pain symptoms in fibromyalgia (FM). However, little is known about the neurophysiological mechanisms mediating these effects. In this study we investigated resting-state connectivity using functional magnetic resonance imaging (fMRI) before and after a 15 week standardized exercise program supervised by physical therapists. Our aim was to gain an understanding of how physical exercise influences previously shown aberrant patterns of intrinsic brain activity in FM. Fourteen FM patients and eleven healthy controls successfully completed the physical exercise treatment. We investigated post- versus pre-treatment changes of brain connectivity, as well as changes in clinical symptoms in the patient group. FM patients reported improvements in symptom severity. Although several brain regions showed a treatment-related change in connectivity, only the connectivity between the right anterior insula and the left primary sensorimotor area was significantly more affected by the physical exercise among the fibromyalgia patients compared to healthy controls. Our results suggest that previously observed aberrant intrinsic brain connectivity patterns in FM are partly normalized by the physical exercise therapy. However, none of the observed normalizations in intrinsic brain connectivity were significantly correlated with symptom changes. Further studies conducted in larger cohorts are warranted to investigate the precise relationship between improvements in fibromyalgia symptoms and changes in intrinsic brain activity.

## Highlights

• Physical exercise reduced the impact of fibromyalgia (FM) in everyday life.
• Longitudinal changes included restored insular-sensory functional connectivity.
• The links between connectivity and symptom improvements require further studies.

## 1 Introduction

Fibromyalgia (FM) is a condition characterized by widespread chronic pain and is often accompanied by cognitive dysfunction and fatigue. The pathogenesis is still largely unknown, but there is evidence of both peripheral and central pathophysiology (for a review, see Clauw, 2014). The diagnosis is currently based on self-reported pain, and as of yet, no laboratory test can directly test for FM. Intriguingly however, several brain imaging studies of FM patients indicate an altered functional brain structure which is related to aberrant pain evoked brain activation, particularly in the anterior cingulate cortex and thalamus (Jensen et al., 2013). Resting state brain connectivity constitutes a promising complement to the traditional task-based fMRI studies employing subtraction designs that disregard the brain activity reflecting sustained pain states. Resting state fMRI studies have reported altered intrinsic brain activity in FM patients including: decreased connectivity between insula and prefrontal areas (Ichesco et al., 2014) and the periaqueductal grey (PAG) (Pujol et al., 2014), increased connectivity between insula and medial regions of the default mode network (DMN) (Napadow et al., 2010), and decreased connectivity between somatosensory regions and visual and auditory cortices (Pujol et al., 2014). Using a comprehensive set of analytical approaches to characterize intrinsic brain activity in FM, we recently showed a decreased connectivity between pain-related and sensorimotor brain areas during rest (Flodin et al., 2014).

Physical exercise is a potent treatment for FM, on par with the efficiency of cognitive behavioural therapy, education in coping strategies and pharmacological interventions (Clauw, 2014). In this study, we aimed to investigate if physical exercise could normalize the previously described aberrant patterns of intrinsic brain connectivity in FM and if this was related to symptom improvement. To our knowledge, this is the first study to investigate the longitudinal effects of physical exercise on intrinsic brain activity in FM. Our hypothesis was that the physical training program would influence the previously detected deviant connectivity patterns in six pairs of brain ROI-to-ROI (Region-of-Interest) connectivity in FM patients (Flodin et al., 2014). We further aimed to investigate the extent to which longitudinal changes in connectivity correlated with changes in symptoms.

## 2 Methods

### 2.1 Subjects

Subject recruitment and inclusion criteria were identical to our baseline study (Flodin et al., 2014). All FM patients satisfied the American College of Rheumatology (ACR) 1990 disease criteria for FM. Of the 16 female subjects included in the baseline study, two subjects failed to comply with the longitudinal study protocol. The mean age of the remaining fourteen subjects was 48.4 (range 25–64) years (all female). The mean symptom gravity (assessed with the Fibromyalgia Impact Questionnaire (FIQ), see Bennett, 2005 for reference) was 60.8 (*SD* = 11.8), and the mean FM duration was 7.3 years (*SD* = 4.0). Among the 22 female subjects originally included as healthy controls in the baseline study, nine subjects were only scanned once, fMRI data from one subject was discarded due to excessive head-motion, and fMRI data from one subject was rejected due to technical failures in the image acquisition process. Thus, fMRI resting-state data from eleven healthy controls were included (mean age 41.8 years, range 20–63).

### 2.2 Training intervention

A 15-week exercise program with two sessions each week was carried out under supervision from a physiotherapist (PT). Before the participants started the intervention, they had individual meetings with a PT who tested their one repetition maximum (1 RM) and tolerance before deciding the initial load of each exercise. At the same time the participants received individual instructions for each exercise. Each session lasted for about 1 h and included 10 min of warming up by ergometer cycling, isometric exercises for the deep muscles in the back and stomach, and concentric and non-concentric exercises for the legs, back, stomach, arms and hands. The program ended with stretching exercises.

The participants' individual 1 RM for the different exercises were tested before starting and at three time points during the program. For the legs and arms the initial loads were set at approximately 40% of one 1 RM and the participants were instructed to repeat each exercise 15–20 times in one to three sets within symptom tolerance. In between each set, the participants rested for at least 45 s. After 5 weeks the load was raised up to about 50% of 1 RM with 2 sets of 12–15 repetitions, after 8 weeks up to about 60% of 1 RM with 2 sets of 10–12 repetitions and after 12 weeks up to about 70% of 1 RM with 2 sets of 8–10 repetitions. Leg exercises for explosive strength were also included at weeks five and eight.

The participants reported pain and other adverse effects of the exercise program to the PT during every session. In the case that the level of pain increased without returning back to normal within a few days, the participants were instructed to lower the load, but continue to do the exercise. The same instructions were given if the participants had a bad day or an increase of symptoms. If any special exercise was causing problems the participants were instructed to refrain from doing it. The PT followed up on program compliance; if participants did not participate in a session they were instructed to give notice with the reason of absence, on which the physiotherapist made a follow-up phone call. The overall compliance rate was high, with an average of 29.12 (*SD* = 3.2) training sessions taken for the 25 participants (for FM: *M* = 29.4, *SD* = 1.6; HC: *M* = 28.7, *SD* = 4.5). There was no significant group difference with regard to compliance (*t*(23) = 0.52, *p* = .60).

### 2.3 Behavioural measures

We used two behavioural measures to estimate pain and fibromyalgia symptomatology. We used the bodily pain subscale of the Short Form Health Survey (SF36BP) for pain assessment, since this assesses pain during a 4 week period and thus reflects long-term changes, disregarding temporary fluctuations in pain intensity (Contopoulos-Ionannidis et al., 2009). In addition, we used the Fibromyalgia Impact Questionnaire (FIQ), which is a general questionnaire regarding the impact of fibromyalgia on everyday life (Bennett, 2005).

### 2.4 MRI data acquisition

Anatomical and functional MR imaging were performed on a 3 T General Electric 750 MR scanner. For each subject we performed one resting state scan consisting of 200 volumes, using an echo-planar imaging sequence with TR/TE = 2500/30 ms, flip = 90°, 49 slices, 96 × 96 matrix size, FOV = 288×288 mm, slice thickness = 3 mm using interleaved slice acquisition. In the resting state condition, subjects were instructed to lie still and rest, and keep their eyes closed and try not to fall asleep. Prior to the resting state fMRI data acquisition, subjects underwent two fMRI sessions of a task-evoked pain fMRI paradigm (approx. 7 min each), and two sessions of an fMRI adopted version of the Stroop task (approx. 7 min each). Data from the task-evoked fMRI sessions will be reported elsewhere.

### 2.5 Resting state fMRI data analysis

**Supplementary Fig. S1:**
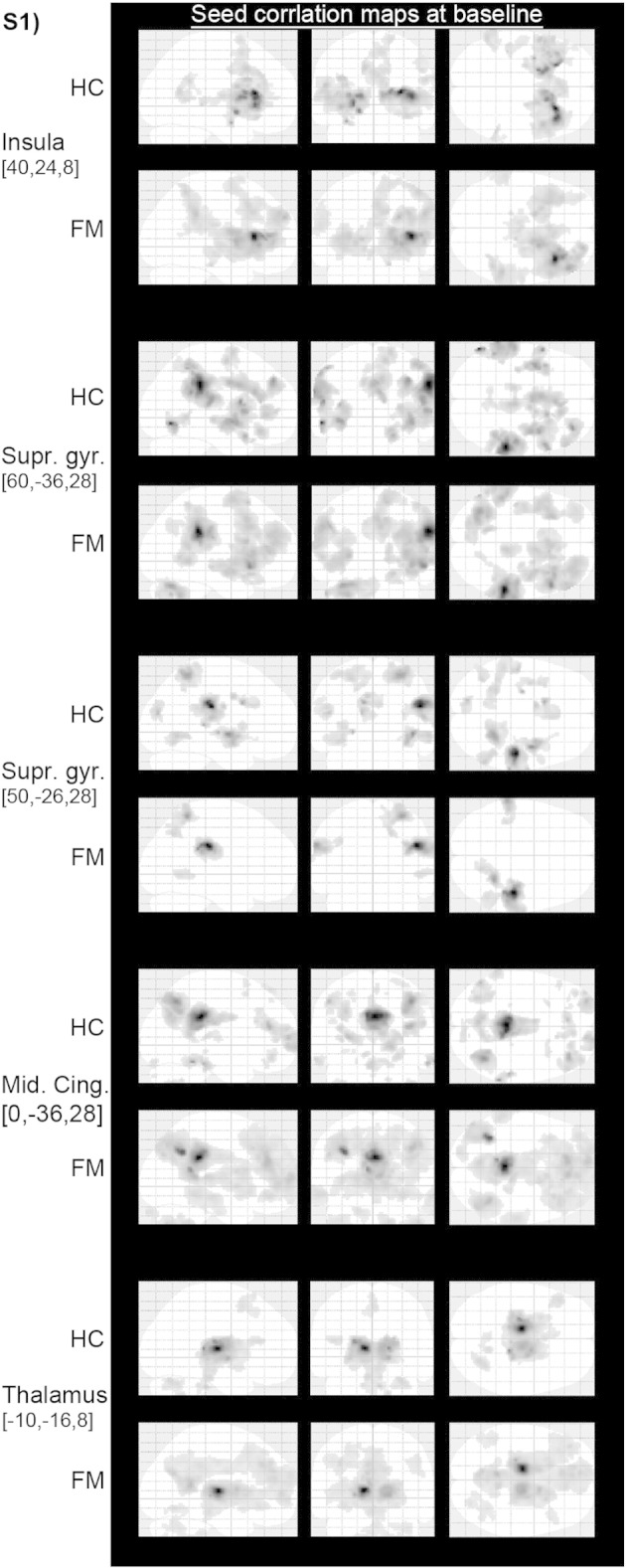
Whole brain connectivity maps of the five investigated seed regions at baseline. Maps are thresholded at cluster level significance of p < 0.05 FWE, with a cluster defining voxel threshold of p < 0.001.

In this study we investigated longitudinal changes (post- versus pre-physical exercise treatment) of functional connectivity of six seed regions located in pain regions as demarked by a metaanalysis on 314 pain studies carried out in the framework of neurosynth (Yarkoni et al., 2011), that we previously found to be affected by FM in our baseline study (FM: n = 16, HC: n = 22, see Flodin et al., 2014). Specifically, the spherical Region-of-Interest (ROI, with a radius of 4 mm) were located in the insula, sensorimotor cortex and thalamus. A detailed list of the anatomical location of the five seed ROIs and the corresponding six target clusters is given in Table 1. Supplementary Fig. S1 illustrates the whole brain connectivity maps pertaining to each of the seed regions shown at baseline for both the FM and the HC cohort.

Image preprocessing, seed-based correlation analysis (SCA) and group-level analyses were carried out in Matlab (Mathworks Inc., Natick, MA, USA). Prior to SCA, imaging data were preprocessed using the Matlab toolbox SPM12 (Wellcome Trust Centre of Neuroimaging, University College London, UK). Image preprocessing included slice time correction to the middle slice, realignment to the mean image using the 4th degree of B-spline interpolation, co-registration of functional and structural images, tissue segmentation of structural images, normalization of structural and functional scans to the MNI template using the deformation field obtained from the segmentation (4th degree B-spline function, resampling to 2 mm isotropic voxels). Finally, functional volumes were spatially smoothed using an 8 mm FWHM Gaussian kernel. Subject level SCA analyses were carried out using the Conn toolbox (http://www.nitrc.org/projects/conn) (Whitfield-Gabrieli & Nieto-Castanon, 2012). Functional volumes were band pass filtered at 0.008–0.09 Hz (default values). Subject specific nuisance regressors included 6 movement and their time derivatives, and 5 regressors pertaining to white matter and CSF signals respectively, using a component based noise correction (CompCor) approach (Behzadi et al., 2007). Additionally, images that were regarded as movement outliers were regressed out. Outliers were detected using the ART toolbox (http://nitrc.org/projects/artifact_detect/) and defined as volumes with frame wise displacement (FD) larger than 0.5 mm or signal intensity changes greater than 3 standard deviations (default thresholds). For both pre- and post-treatment fMRI data, estimates for the strength of resting-state functional connectivity were obtained from each subject and for each of the six ROI-to-ROI connectivity pairs.

The computed estimate of the strength of connectivity (β values) between the seed ROIs and target clusters was subsequently used at a second level group model of differences in intrinsic connectivity (Δβ_FM_ and Δβ_HC_) (after controlling for age and inter-individual differences in mean frame-wise displacement, see Flodin et al., 2014). Additionally, we tested for the relationship between changes in functional connectivity and changes in behaviour (post-minus pre-physical exercise treatment), using the Pearson correlation coefficient. Since we were interested in connectivity changes that were specific to the FM condition (rather than general training effects independent of group), we compared the post- versus pre-change in intrinsic brain connectivity in the FM patients versus the HC cohort (Δβ specificity = |Δβ_FM_ − Δβ_HC_|). Due to relatively unequal group sizes (14 FM and 11 HC), we conducted permutation tests that are less sensitive to unbalanced designs compared to parametric t-tests. Thus, for each of the six seed-target connectivity pairs, we estimated the likelihood of the observed group difference, given the null hypothesis that there would be no difference in longitudinal change in connectivity, by randomly shuffling the group membership of the subjects (1000 permutations, p-value threshold <0.05, Bonferroni corrected).

## 3 Results

### 3.1 Behaviour

**Fig. 1:**
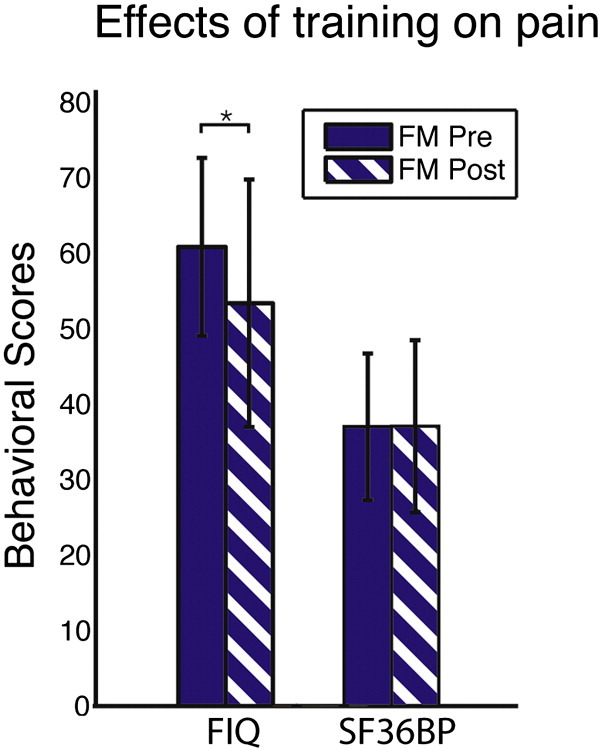
Average FIQ and SF36BP ratings in 14 FM patients before (solid bars) and following (striped bars) the exercise intervention. The reduction in FIQ ratings indicates reduced FM symptoms. No change was observed in pain ratings (SF36BP). The asterisk sign (*) signifies a significant difference at p < 0.01 between post- versus pre-treatment conditions. Error bars denote standard deviations. FIQ = Fibromyalgia Impact Questionnaire, SF36BP = short form bodily pain subscale.

Pre-treatment, the mean SF36BP score for the FM cohort was 37.00 ± 9.70, (highest subjective health, i.e. no pain = 100) whereas the post-treatment score was 37.07 ± 11.40 (*t*(14) = −0.026, *p* = 0.98). Further, in the FM cohort, the pre-treatment mean FIQ was 60.8 ± 11.8 (maximally severe FM impact = 100), and for post-training 53.3 ± 29.5 (*t*(13) = 2.282, *p* = 0.040, effect size *r* = 0.53). The FIQ and SF36BP were negatively correlated both pre- (*r* = −0.78, *p* = 0.00080) and post-treatment (*r* = −0.79, *p* = 0.00089) (Fig. 1).

### 3.2 Intrinsic brain connectivity

**Fig. 2:**
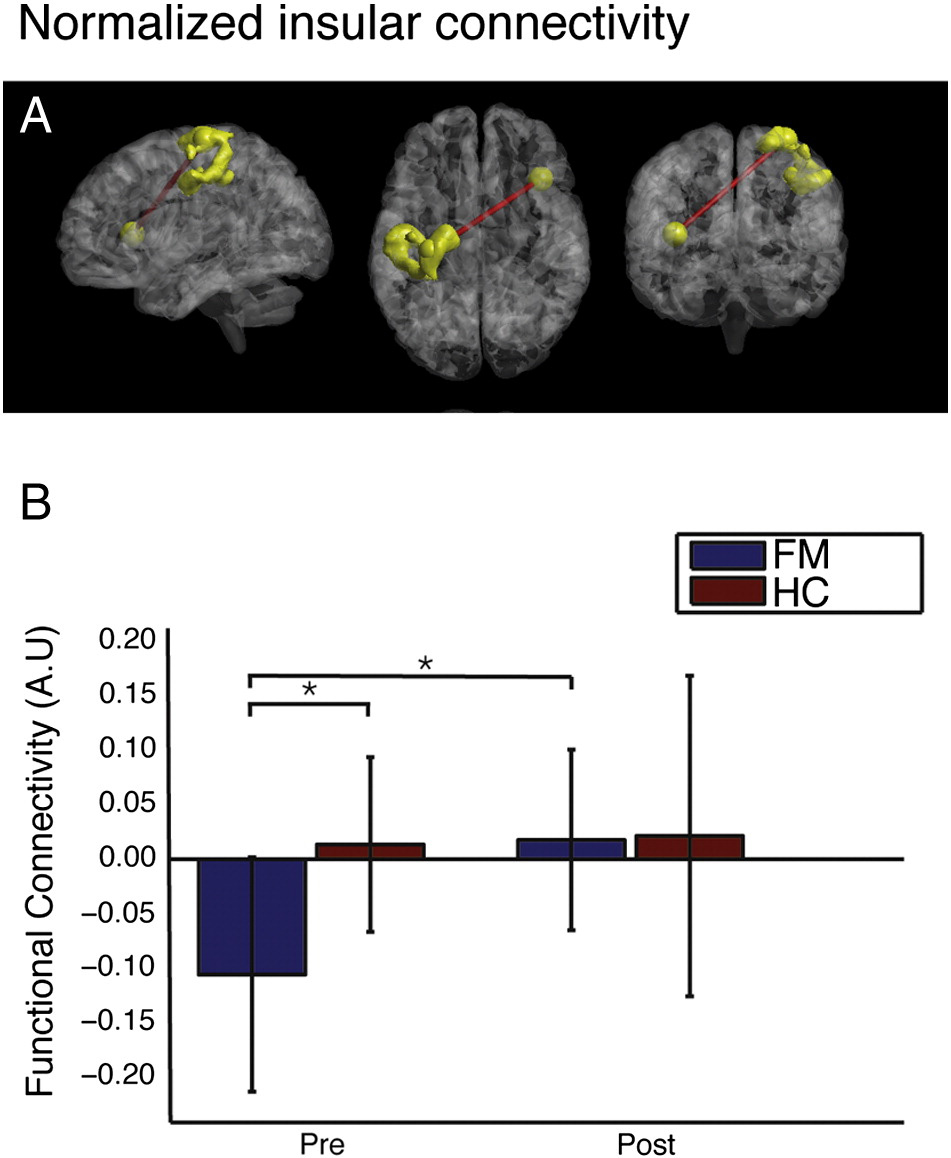
Physical exercise induced normalization of resting state connectivity between the right insula and the left sensorimotor region in the FM cohort. (A) Intrinsic connectivity between a spherical seed region (radius = 4 mm) located in the right anterior insula and a cluster extending 1490 voxels in the left sensorimotor cortex. (B) Post- versus pre-treatment insular-sensorimotor connectivity (arbitrary units) for fibromyalgia (blue) and controls (red). Error bars denote standard deviations.

Out of the six seed connectivity pairs tested, four showed a significant normalization among the FM patients (see Table 1). The observed changes included an increased connectivity between the right anterior insula and left primary sensory motor areas, the right supramarginal gyrus and primary sensorimotor areas, and the right supramarginal gyrus and left inferior prefrontal cortex. The aberrantly high baseline connectivity in FM between supramarginal gyrus and cerebellum was normalized. Noteworthy, also healthy controls showed significant longitudinal connectivity changes. Hence, in order to investigate the extent to which the change in intrinsic connectivity was selective to the FM group, we performed permutation tests by which the absolute difference in magnitude of the connectivity change between the two groups was compared. In the permutation test, only the change in connectivity between the insula and primary sensorimotor regions showed a selective normalization in the FM group (see Table 1 and Fig. 2). None of the treatment-related changes in intrinsic connectivity correlated significantly with the FIQ or SF36BP scores (Table 1).

## 4 Discussion and conclusion

In this study we have shown that the previously reported abnormal patterns of resting state connectivity in FM patients are partly normalized after a 3 month schedule of regular physical exercise. Although several intrinsic brain connectivity patterns underwent longitudinal change, only the connectivity between the right insula and primary sensorimotor cortex displayed a selectively greater change for FM compared to HC. It should be noted that all the seed regions used in the current study belongs to a set of brain areas that commonly are activated in pain studies, as delineated by an automated meta-analysis (described in Flodin et al., 2014). The insula plays a pivotal role in pain processing (Duerden et al., 2013) and activity in its anterior part has been associated with interoceptive accuracy and subjective feeling (Critchley et al., 2004). Interestingly, a recent study in FM patients reported a hypo-connectivity between the bilateral anterior insula and PAG (peri-aqueductal grey nucleus), that correlated with symptom severity (Pujol et al., 2014). Speculatively, the shift towards increased insula connectivity following physical exercise therapy observed here could reflect an increased interaction between brain regions responsible for interoceptive valiance and bodily representations. The restoration of functional connectivity between the right SMG and primary sensorimotor areas, as well as between the right SMG and inferior prefrontal cortex, consisted in reduction of anticorrelations for FM patients.

Whereas functional connectivity is commonly interpreted as (at least partly) reflecting a history of co-activation of brain regions interacting in order to accomplish a specific (cognitive or housekeeping) function at a given time, the functional significance of anticorrelation is less well understood. It has previously been shown that anticorrelations can be artificially induced during preprocessing of the data, such as by global mean regression of the fMRI signal intensity time-series (Murphy et al., 2009). However, using electrophysiological techniques, a neurobiological origin of anticorrelations has been confirmed (Keller et al., 2013). Furthermore, preprocessing strategies that omit global signal regression, including the CompCor strategy that is employed here, commonly render anticorrelations (Chai et al., 2012). The general functional significance of anticorrelations, especially outside the well studied relationship between the default mode network and task positive networks (Fox et al., 2005; Fransson, 2005), remains tentative. One interpretation of the normalization (i.e. decreases in anticorrelation) observed here, is that pain- and somatosensory regions interact in a more coherent fashion, implying a reduced mismatch between sensorimotor signals and activity in brain regions involved for pain processing. Our finding complement results from previous FM intervention studies that primarily reported decreased insular connectivity following FM interventions. For instance, Schmidt-Wilcke et al. (2014) found that improvements in clinical pain correlated with decreased functional connectivity between the anterior insula and ACC following a 6 week long milnacipran treatment. Likewise, the same research group observed a decreased connectivity between insula and the default mode network following an acupuncture treatment (Napadow et al., 2012). These results were interpreted as a restoration of FM-associated hyperconnectivity between the brain regions involved in pain perception (anterior insula) and self-referential thought (DMN). Thus, an emerging picture is that improvements in symptom severity in FM could be associated either with reduced interaction between regions sub-serving pain processing and self-related cognition, or by a decreased asynchrony between the pain and sensorimotor regions. Since the current study investigated the effects of physical exercise, it is plausible that the mechanism mediating these symptom improvements to a larger degree involves the sensorimotor brain areas.

Few studies have directly investigated longitudinal changes of intrinsic brain connectivity following physical exercise interventions. Becerra et al. (2014) investigated the effects of a three week physical and psychological training program in a group of children and adolescents with paediatric complex regional pain syndrome. They reported treatment effects in several networks (e.g. DMN, salience networks and cerebellum). However, significance levels were not adjusted for testing of multiple networks, and their relatively small cohort (12 patients and an equal number of controls), prevents any strong conclusions on the effect of the intervention. There are several studies on effects of motoric skill training, such as sequential motor training paradigms with durations of a couple of weeks. A general finding among these studies is that an initially increased task evoked responses in both the primary sensory and primary motor cortices (Floyer-Lea & Matthews, 2005; Hlustík et al., 2004). Interestingly, also resting state regional cerebral blood flow (rCBF, measured with positron emission tomography) in the primary motor cortex is increased following motor training (Xiong et al., 2009). Focusing on resting state connectivity, Krafft et al. (2014) studied the effects of an 8 month exercise intervention in a cohort of overweight children. They report increased network synchronicity within the motor network, and decreased synchronicity within the DMN and cognitive control networks in the intervention group relative to controls. Another study reports on the acute effect of a 20 minute aerobic exercise intervention on resting state activity (Rajab et al., 2014). Their primary finding was an increased co-activation within the primary and sensorimotor areas.

Although the current study is the first to investigate the resting state changes following an exercise intervention among fibromyalgia patients, there are several studies coherent with our finding that physical exercise primarily influences functional connectivity within cardinal sensorimotor regions.

The beneficial health effects of physical exercise are well acknowledged. Following the intervention, FM patients rated lower on the FIQ scale, reflecting increased healthiness and a decrease of symptom severity, physical disability and overall impact of fibromyalgia. However, the body pain levels (SF36BP) did not change. These findings indicate that the therapeutic effects of the 3 month exercise intervention rendered improvements of symptoms that only indirectly pertained to pain. Thus, the observed normalizations of functional connectivity involving sensory areas could possibly reflect subclinical therapeutic processes, likely induced by increased levels of motor activity.

However, since neither SF36BP nor FIQ could be linearly correlated with change in brain activity in the current study, the behavioural implications of the observed normalization in intrinsic connectivity warrants further investigation. A possible explanation for the absent brain–behavioural coupling could be that treatment-induced changes in brain connectivity follow an inverted u-shape temporal profile as proposed by the expansion–partial normalization hypothesis of neuroplasticity (Brehmer et al., 2014). However, plotting change in behavioural scores against change in connectivity did not indicate any such relationship. Another possible scenario is that the behavioural parameters monitored in the present investigation were subserved by different neuronal processes other than the ones investigated here.

We note several limitations in the current study. First, the resting state scans were acquired approximately 30 min after two task fMRI sessions were administered in the MR scanner (Stroop and evoked pain, data will be reported elsewhere). Thus, we cannot rule out the possibility that putative spill-over effects from task-based fMRI paradigms occur, which could have had a differential influence on the group brain activity patterns. Second, since our study did not include a placebo or no-treatment FM control group, the observed longitudinal changes in brain connectivity might in part be driven by spontaneous improvements in pain severity and accompanying changes in brain connectivity that is not attributable to the physical intervention per se (i.e. “regression towards the mean”, Stigler, 1997). Third, changes in intrinsic brain connectivity might be unrelated to the measured improvements in pain (at least in relation to the pain measures used here). Theoretically, the observed connectivity changes could be accompanied by changes in other commonly reported FM symptoms such as mood changes, insomnia, fatigue or impairments in cognitive function, although the fact that the seed regions were placed in pain-related brain areas supports the interpretation that there is a link between the physical intervention and pain relevant brain processes. Finally, we suggest that our findings should be treated to some extent as preliminary in nature, given the relatively small number of participants that completed the post-therapy imaging session. Future studies using larger study samples would likely enable a more detailed investigation of the relationship between changes at the neurophysiological versus the behavioural level. Additionally, it might be desirable for future studies to record brain connectivity and behaviour at multiple time-points and thereby achieve a temporally more fine-grained characterization of the longitudinal neurophysiological and behavioural changes related to exercise therapy in FM patients.

In conclusion, we have shown that a therapeutic physical exercise-training program increases the perceived degree of healthiness in patients diagnosed with fibromyalgia. Interestingly, we observed a restoration of functional connectivity between several pain and sensorimotor regions. In this context, it is important to note that longitudinal change of intrinsic connectivity between the sensorimotor system and the insula was observed exclusively in the FM cohort. This finding suggests that the insula connectivity may play a key role for the observed physical therapy-induced effects on FM symptomatology.

The following is the supplementary data related to this article.

## Conflicts of interest

None declared.

## Funding

This study was supported by grants from the Swedish Rheumatism Association, Stockholm County Council - 2010, Swedish Foundation for Strategic Research (2012-0179), Swedish Research Council K2013-52X-22199-01-3, and Karolinska Institute Foundation (2012FoBi34779). The funders had no role in the study design, data collection and analysis, decision to publish, or preparation of the manuscript.

## Figures and Tables

**Table 1:** 

Functional connectivity (seed) ↔ (target)	Δβ_FM_ t (p)	Δβ_HC_ t (p)	Δβ specificity |Δβ_FM_ − Δβ_HC_ | t (p)	β_HC_ > β_FM_ baseline t (p)	Correlation of Δβ_FM_ and FIQ r (p)
Insula (40, 24, 8) ↔ S1/M1 (−30, −22, 68)	3.95 (.0017)*	0.133 (.90)	0.12 (.0050)*	3.064 (.0055)*	0.019 (.95)
Supr. gyr. (60, −36, 28) ↔ S1/M1 (10, −24, 80)	3.12 (.0081)*	−0.98 (.35)	0.023 (0.48)	3.12 (.0048)*	0.14 (.63)
Supr. gyr. (50, −26, 28) ↔ inferior PFC (−32, 30, −8)	3.34 (.0053)*	−2.23 (.050)	0.0041 (.86)	4.063 (.00048)*	0.49 (.076)
Mid cing (0, −36, 28) ↔ occipital (42, −72, −12)	1.94 (.074)	0.41 (.69)	0.089 (.030)	2.58 (.016)	0.16 (.59)
Thalamus (−10, −16, 8) ↔ premotor (0, 6, 56)	1.44 (.17)	−0.20 (.85)	0.049 (.21)	2.30 (.031)	−0.28 (.32)
Supr. gyr. (60, −36, 28) ↔ cerebellum (−42, −74, −34)	−3.60 (.0033)*	3.45 (.0062)*	0.03 (.38)	−6.1 (.0000035)*	0.21 (.47)

Summary of changes in intrinsic brain connectivity related to physical exercise training and correlations between intrinsic connectivity and the only pain-related parameter that displayed significant longitudinal changes (FIQ) among FM patients.

Out of the six pairs of brain regions tested, four displayed a significant longitudinal renormalization in the fibromyalgia cohort. The degree of connectivity was significantly changed for the healthy control between the supramarginal gyrus and cerebellum. Of note, only the change of connectivity between the insula and S1/M1 was relatively significantly larger for FM than that for HC (no change in connectivity was significantly larger for the healthy compared to fibromyalgia). The level of statistical significance (p = 0.05) was corrected for multiple comparisons with regard to the six tested seed regions using Bonferroni correction (p < 0.0083). Statistical significant changes are marked with “*”. Abbreviations: Supr. gyr. = supramarginal gyrus; S1/M1 = primary sensorimotor areas; ΔB = changes in connectivity; t = t-value; p = p-value; *r* = Pearson correlation coefficient. All coordinates (x, y, z) are in MNI space.
